# Use of nitrous oxide as a purge gas for automated nitrogen isotope analysis by the Rittenberg technique

**DOI:** 10.1155/S1463924697000175

**Published:** 1997

**Authors:** R. L. Mulvaney, S. A. Khan, G. K. Sims, W. B. Stevens

**Affiliations:** 1 Department of Natural Resources and Environmental Sciences University of Illinois Urbana Illinois 61801 USA; 2 USDA-ARS Urbana Illinois 61801 USA; 3 Department of Crop Sciences University of Illinois Urbana Illinois 61801 USA; 4 Department of Natural Resources and Environmental Sciences Turner Hall 1102 South Goodwin Avenue Urbana Illinois 61801 USA

## Abstract

An apparatus that operates with an isotope-ratio mass spectrometer to automatically perform nitrogen isotope analyses by the Rittenberg technique was modified to permit the use of nitrous oxide (N_2_O) instead of Freon (CCl_2_F_2_ or CHClF_2_) for the purging of air prior to hypobromite oxidation of ammonium-N to N_2_ in a plastic microplate. Analytical performance was unaffected by the modifications. Up to 768 samples can be processed in a single loading, at a rate of 6 to 12 samples/h. Within the range of 0.2 to 20 atom % ^15^N, isotope-ratio analyses of 50 to 200 μg of N using the automated Rittenberg apparatus (ARA) with a double-collector mass spectrometer were accurate to within 0.7%, as compared to manual Rittenberg analyses of 1 mg of N using the same mass spectrometer with a dual-inlet system. Automated analyses of 20μg of N were accurate to within 2%, and automated analyses of 10 μg of N were accurate to within 7%. The relative standard deviation for measurements at the natural abundance level (10 analyses, 20-200 μg of N) was < 0.04 %.


Journal of Automatic Chemistry, Vol. 19, No. 5 (September–October 1997) pp. 165–168

### Use of nitrous oxide as a purge gas for automated nitrogen isotope analysis by the Rittenberg technique

**R. L. Mulvaney, S. A. Khan**

*Department of Natural Resources and Environmental Sciences, University of Illinois, Urbana, Illinois 61801, USA*

**G. K. Sims**

*USDA-ARS, Urbana, Illinois 61801, USA*

**and W. B. Stevens**

*Department of Crop Sciences, University of Illinois, Urbana, Illinois 61801, USA*

*An apparatus that operates with an isotope-ratio mass spectrometer to automatically perform nitrogen isotope analyses by the Rittenberg technique was modified to permit the use of nitrous oxide ($N_2O$) instead of Freon ($CCl_2F_2$ or $CHClF_2$) for the purging of air prior to hypobromite oxidation of ammonium-N to $N_2$ in a plastic microplate. Analytical performance was unaffected by the modifications. Up to 768 samples can be processed in a single loading, at a rate of 6 to 12 samples/h. Within the range of 0·2 to 20 atom % $^{15}N$, isotope-ratio analyses of 50 to 200 µg of N using the automated Rittenberg apparatus (ARA) with a double-collector mass spectrometer were accurate to within 0·7%, as compared to manual Rittenberg analyses of 1 mg of N using the same mass spectrometer with a dual-inlet system. Automated analyses of 20 µg of N were accurate to within 2%, and automated analyses of 10 µg of N were accurate to within 7%. The relative standard deviation for measurements at the natural abundance level (10 analyses, 20–200 µg of N) was $<$ 0·04%.*

### Introduction

Much progress has been made during the past decade in the automation of N isotope analyses for agricultural and environmental research. The most common approach has been to interface a CN elemental analyser to an isotope mass spectrometer [1, 2]; the combination is referred to as an ANCA-MS (for automated N/C analyser-mass spectrometer), or CF-IRMS (for continuous flow-isotope ratio mass spectrometer). During analyses, N in the sample is converted to $N_2$ by combustion at approximately 1700 °C, and a small fraction of the $N_2$ is admitted to an isotope mass spectrometer for measurement of the ion currents at $m/z$ 28, 29 and 30, from which total N content and atom % $^{15}N$ are determined.

The Rittenberg technique has also been utilized in the automation of mass spectrometers for N isotope analysis [3–6]. In this technique, $N_2$ is generated from ammonium ($NH_4^+$), through reaction with alkaline hypobromite in the absence of air [7, 8]. For total $^{15}N$ analysis, conversion of sample N to $NH_4^+$ is done by the Kjeldahl method, which involves digestion with concentrated $H_2SO_4$ to convert organic forms of N to $NH_4^+$-N, followed by steam distillation or diffusion of the digest with alkali [8, 9]. Alternatively, the Rittenberg technique may be employed for $^{15}N$ analysis of specific forms of N, such as $NO_3^-$, $NO_2^-$, α-amino acids, or amino sugars [10, 11].

Although the automated Rittenberg apparatus (ARA) may appear to be at a considerable disadvantage relative to an ANCA-MS because of the need for wet chemical processing to convert sample N to $NH_4^+$-N, a method of automation based on the Rittenberg technique has several advantages for N isotope analysis. The ARA provides greater throughput capacity than can be achieved with an ANCA-MS, at a lower cost per analysis. Moreover, analyses with the ARA can be performed over an exceptional range of sample N content, from 10 µg to more than 1 mg [6], owing to the use of a pressure transducer for inlet pressure regulation [3, 5, 6]. Of particular significance is that simple diffusion methods using an $H_3BO_3$-indicator solution may be employed to speciate inorganic N in water or soil extracts for isotope-ratio analysis by ARA-MS [11, 12]. These methods permit quantitative determinations by acidimetric titrimetry, as well as N isotope analyses; whereas, with ANCA-MS, diffusions to speciate inorganic N for isotope analysis are done using an acidified filter disk to collect the diffusing $NH_3$ [13, 14], and a separate analysis is necessary to determine the inorganic N concentration of the sample, usually involving an automated colorimetric analyser.

In the original development of the ARA [3], Freon-12 ($CCl_2F_2$) was employed as a liquid $N_2$-condensible purge gas. The same gas was used in early work with the commercial version of the ARA [5], but was subsequently replaced by Freon-22 ($CHClF_2$) [6] because of restrictions on the consumption of Freon-12 arising from international concern over depletion of stratospheric ozone and global warming by chlorofluorocarbon (CFC) refrigerants. In accordance with the Clean Air Act Amendments of 1990, production of Freon-12 ceased in the United States on 1 January 1996. Freon-22 remains available, but like other hydrochlorofluorocarbon (HCFC) refrigerants, is subject to gradual phasing out from 2004 [15].

Besides the adverse environmental implications, and the problem of declining availability, commercially available Freon is contaminated with air and must be purified before being used as a purge gas for $^{15}N$ analyses by ARA-MS [5, 6]. Moreover, the refrigerant-grade material may have a substantial hydrocarbon content. The latter problem became so serious in the authors' laboratory as to necessitate a substitute for Freon, and ulti-

Correspondence to R. L. Mulvaney, Department of Natural Resources and Environmental Sciences, Turner Hall, 1102 South Goodwin Avenue, Urbana, Illinois 61801, USA.

0142-0453/97 $12·00 © 1997 Taylor & Francis Ltd mately led to the use of $N_2O$ as a purge gas for isotope-ratio analyses by ARA-MS. This paper describes the modifications that permit this change and provides an evaluation of analytical performance.

### Experimental

### *Hardware*

The ARA used is a prototype unit that was developed in collaboration with the Premier American Technologies Corporation (formerly Measurement and Analysis Systems, and Nuclide Corp.), Bellefonte, Pennsylvania. (Names are necessary to report factually on available data; however, the USDA neither guarantees nor warrants the standard of the product, and the use of the name by USDA implies no approval of the product to the exclusion of others that may also be suitable.) This unit was operated with a double-collector mass spectrometer (Nuclide Model 3-60-RMS) [5], which is also equipped with a manually operated dual-inlet system [6].

Detailed descriptions of the design and components of the ARA have been published previously [5, 6]. Three hardware modifications were necessary to permit the use of $N_2O$ as a purge gas for isotope-ratio analyses with the ARA. The U-type cold trap originally employed was redesigned to increase efficiency. The increase was achieved with a three-stage design, in which gas flow first occurs through approximately 4 in of 1/8th in o.d. (0·083 in id) stainless-steel tubing, then through 2 in of the same tubing which had been flattened to reduce the id to 1/16th in, and, finally, through a 6-in coil of 1/16th in od (0·030 in id) stainless steel tubing that was silver-soldered into the flattened section of 1/8th in od tubing. To reduce the velocity of gas flow and thereby increase trapping efficiency, a short section of stainless-steel capillary tubing (0·063 in od, 0·016 in id) was connected between the drawback valve and the cold trap. Finally, the plungers used in all solenoid valves under vacuum were cross-drilled to eliminate hold-up of $N_2O$ during heating of the cold trap.

### *Software*

The software used to operate the ARA was modified to reduce contamination by atmospheric $N_2$ during hypobromite oxidation of $NH_4^+$ salt samples, maximize the efficiency of $N_2O$ trapping and evacuation, and monitor the presence of $N_2O$ in the mass spectrometer during isotope-ratio analyses. A further modification was made to automatically decrease the sample inlet pressure in the event of numerator overflow during isotope-ratio analysis, and to carry out the analysis once the pressure has been decreased sufficiently to eliminate the overflow. The latter capability allows isotope-ratio analyses to be performed regardless of the $^{15}N$ concentration.

### *Nitrous oxide*

The $N_2O$ used was a semiconductor-grade gas (ULSI purity) obtained from Matheson, Cucamonga, California, that was certified to contain $< 2\ \mu l$ of $N_2$ per l.

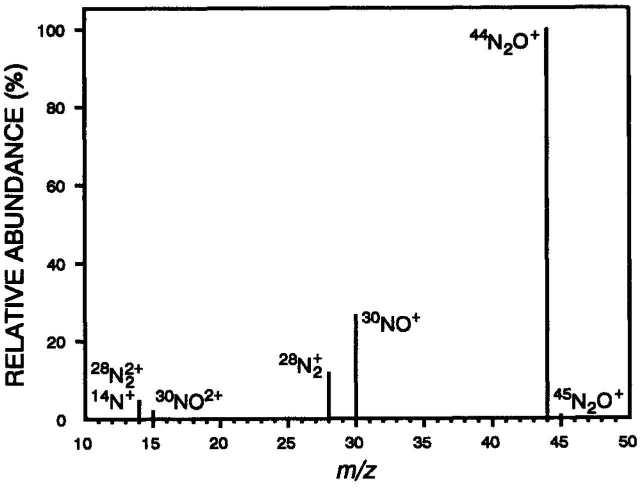

*Figure 1. Mass spectrum of $N_2O$ (electron impact ion source: ionization potential, 70 ev; acceleration potential, 3 kV).*

### Results and discussion

Besides being nontoxic, economical, and of high purity (particularly as regards a low $N_2$ concentration), a purge gas for the ARA must meet three requirements: the gas must have a sufficiently high boiling point as to be completely condensible by liquid $N_2$; the gas must be chemically inert toward alkaline hypobromite; and the gas must be easily pumped under vacuum. The first requirement precludes the use of He, while the second precludes the use of $CO_2$, owing to formation of $Br_2$ through reaction of $H_2CO_3$ with hypobromite [6]. The noble gas, Xe, has all of the necessary physicochemical properties: however, the high cost of this gas is a serious limitation.

Nitrous oxide also meets the requirements of a purge gas for the ARA. Nitrous oxide has the advantage of being commercially available in high-purity grades certified to have a low content of $N_2$, whereas commercial Freon is contaminated with air, and purification is essential before use [5, 6]. And, although more expensive than Freon, high-purity $N_2O$ is far less expensive than Xe. The main disadvantage of using $N_2O$ is that, unlike Freon or Xe, the purge gas will be a potential source of interference in the analysis, because $N_2O$ decomposes in the ion source of the mass spectrometer to form $N_2$ and NO, and thereby contributes to the ion currents at $m/z$ 28 and 30. This is illustrated in figure 1, which shows the mass spectrum obtained when $N_2O$ was admitted via the ARA without raising the liquid $N_2$ bath to freeze the cold trap [5, 6].

Initial attempts to use $N_2O$ as a purge gas led to low values in the analysis of $(NH_4)_2SO_4$ standards enriched in $^{15}N$. The source of this difficulty proved to be incomplete trapping of the $N_2O$ by the U-type cold trap originally employed in the design of the ARA, with the result that the measured ratio, $m/z\ 29/(m/z\ 28 + m/z\ 30)$, was reduced by an inflated denominator. To increase the efficiency of trapping, the original cold trap was replaced with one based on a three-stage design, involving a progressive decrease in tubing diameter. Studies showed that a further increase in trapping efficiency could be

*Table 1. Comparison of a manual dual-inlet system and the ARA with $N_2O$ purging for $^{15}N$ analysis of $N_2$ generated from $(NH_4)_2SO_4$.*

| Nominal atom % $^{15}N$ of $(NH_4)_4SO_4$ | Type of analysis | $NH_4^+$-N analysed (μg) | Atom % $^{15}N$ determined ($N$ = 10) | | |
|---|---|---|---|---|---|
| | | | Range | Mean | SD |
| 0·2 | Dual-inlet | 1000 | 0·1910–0·1916 | 0·191 34 | 0·000 19 |
| | ARA | 200 | 0·1910–0·1920 | 0·191 23 | 0·000 30 |
| | | 150 | 0·1910–0·1917 | 0·191 27 | 0·000 23 |
| | | 100 | 0·1908–0·1919 | 0·191 23 | 0·000 35 |
| | | 50 | 0·1903–0·1909 | 0·190 60 | 0·000 19 |
| | | 20 | 0·1940–0·1944 | 0·194 20 | 0·000 23 |
| | | 10 | 0·2025–0·2050 | 0·203 40 | 0·000 76 |
| 0·37 | Dual-inlet | 1000 | 0·3659–0·3660 | 0·365 98 | 0·000 04 |
| | ARA | 200 | 0·3660–0·3664 | 0·366 23 | 0·000 12 |
| | | 150 | 0·3660–0·3664 | 0·366 22 | 0·000 10 |
| | | 100 | 0·3660–0·3663 | 0·366 13 | 0·000 08 |
| | | 50 | 0·3665–0·3667 | 0·366 56 | 0·000 08 |
| | | 20 | 0·3672–0·3676 | 0·367 46 | 0·000 14 |
| | | 10 | 0·3662–0·3729 | 0·369 60 | 0·002 31 |
| 0·5 | Dual-inlet | 1000 | 0·5028–0·5029 | 0·502 86 | 0·000 04 |
| | ARA | 200 | 0·5020–0·5026 | 0·502 33 | 0·000 16 |
| | | 150 | 0·5021–0·5026 | 0·502 33 | 0·000 15 |
| | | 100 | 0·5021–0·5025 | 0·502 31 | 0·000 14 |
| | | 50 | 0·5023–0·5026 | 0·502 42 | 0·000 10 |
| | | 20 | 0·5007–0·5018 | 0·501 21 | 0·000 34 |
| | | 10 | 0·4880–0·4987 | 0·492 70 | 0·003 42 |
| 1·0 | Dual-inlet | 1000 | 1·032–1·034 | 1·0329 | 0·0007 |
| | ARA | 200 | 1·031–1·032 | 1·0312 | 0·0002 |
| | | 150 | 1·031–1·032 | 1·0312 | 0·0003 |
| | | 100 | 1·031–1·032 | 1·0312 | 0·0003 |
| | | 50 | 1·030–1·032 | 1·0308 | 0·0004 |
| | | 20 | 1·016–1·022 | 1·0205 | 0·0017 |
| | | 10 | 0·992–1·014 | 0·9984 | 0·0064 |
| 2·0 | Dual-inlet | 1000 | 1·970–1·971 | 1·9709 | 0·0002 |
| | ARA | 200 | 1·963–1·965 | 1·9636 | 0·0008 |
| | | 150 | 1·963–1·965 | 1·9639 | 0·0005 |
| | | 100 | 1·963–1·967 | 1·9644 | 0·0009 |
| | | 50 | 1·959–1·966 | 1·9630 | 0·0021 |
| | | 20 | 1·937–1·946 | 1·9413 | 0·0027 |
| | | 10 | 1·876–1·896 | 1·8866 | 0·0073 |
| 5·0 | Dual-inlet | 1000 | 5·183–5·189 | 5·1853 | 0·0017 |
| | ARA | 200 | 5·152–5·185 | 5·1635 | 0·0106 |
| | | 150 | 5·159–5·203 | 5·1763 | 0·0142 |
| | | 100 | 5·175–5·213 | 5·1932 | 0·0132 |
| | | 50 | 5·199–5·214 | 5·2050 | 0·0051 |
| | | 20 | 5·082–5·106 | 5·0916 | 0·0086 |
| | | 10 | 4·879–4·981 | 4·9374 | 0·0323 |
| 10·0 | Dual-inlet | 1000 | 10·250–10·267 | 10·2594 | 0·0068 |
| | ARA | 200 | 10·245–10·258 | 10·2505 | 0·0062 |
| | | 150 | 10·245–10·278 | 10·2636 | 0·0127 |
| | | 100 | 10·254–10·312 | 10·2785 | 0·0162 |
| | | 50 | 10·265–10·291 | 10·2793 | 0·0103 |
| | | 20 | 10·242–10·359 | 10·2994 | 0·0309 |
| | | 10 | 10·208–10·336 | 10·2643 | 0·0424 |
| 20·0 | Dual-inlet | 1000 | 19·978–20·006 | 19·9913 | 0·0099 |
| | ARA | 200 | 19·891–19·920 | 19·9004 | 0·0086 |
| | | 150 | 19·887–19·909 | 19·8996 | 0·0100 |
| | | 100 | 19·894–19·912 | 19·9040 | 0·0058 |
| | | 50 | 19·871–19·951 | 19·8923 | 0·0219 |
| | | 20 | 19·726–19·847 | 19·8109 | 0·0394 |
| | | 10 | 19·521–19·712 | 19·5807 | 0·0623 |

achieved by using a capillary tube to reduce the velocity of gas flow through the cold trap, and that an inside diameter of 0·016 in was optimal. Trapping was incomplete with a larger capillary, whereas smaller capillaries led to a serious loss of sensitivity or caused the cold trap to plug, vitiating the analysis.

To check the efficiency of trapping, the software was modified so that the voltage at *m/z* 44 was always measured before isotope-ratio analyses of sample $N_2$; the measured voltage is stored in the memory and printed out on the report. Initial data from these measurements revealed the presence of very little, if any, $N_2O$ during the first analysis in a series, but a substantial presence of $N_2O$ thereafter, sometimes accompanied by serious drift in the ratio measurement. Both problems were eliminated by modifying the software so that evacuation of $N_2O$ during heating of the cold trap was done with the diffusion pump instead of with a rotary pump (as had been the practice with Freon), and by cross-drilling the plungers in all solenoid valves under vacuum to eliminate any hold-up of $N_2O$ in the sample and reference inlet manifolds.

Besides permitting a voltage measurement at *m/z* 44 to check for the presence of $N_2O$, the software was modified so that a voltage was also measured at *m/z* 32, indicating the extent of air contamination. A substantial decrease in the voltage at *m/z* 32, and increased analytical accuracy, was achieved by minimizing the period for reaction of $NH_4^+$-N with LiOBr, which can be attributed to a reduction in the diffusion of air through the polystyrene microplate. This finding motivated a change in software, whereby the final purging with $N_2O$ is performed as late as possible in the operating routine, immediately before the addition of LiOBr. A series of analyses involving different purge times showed that purging is complete when performed for 15 s, given a flow rate of at least 7 ml/s.

To evaluate the accuracy and precision of analyses with the ARA using $N_2O$ as a purge gas, a comparison was made to manual Rittenberg analyses with a dual-inlet system. Eight different $(NH_4)_2SO_4$ solutions were used in this comparison, ranging from 0·2 to 20 atom % in the concentration of $^{15}N$. Analyses with the ARA were performed on 10 to 200 µg of $NH_4^+$-N. Manual analyses were performed on 1 mg of $NH_4^+$-N in a disposable glass vial [8]. Table 1 summarizes the data obtained.

The analytical performance of the ARA with $N_2O$ purging was comparable to that observed in a previous evaluation using Freon-22 [6]. Best results were achieved when automated analyses were performed on 50 to 200 µg of N. In such cases, the difference between any single measurement with the ARA and the mean value obtained with the dual-inlet system did not exceed 0·7%. When automated analyses were performed on 20 µg of N, this difference did not exceed 2%. With 10 µg of N, automated analyses were within 7% of the mean value from manual analyses. Analytical precision tended to be lower with the ARA than with the dual-inlet system. For manual analyses, the relative standard deviation (coefficient of variation) ranged from 0·008 to 0·1%. For automated analyses, the relative standard deviation ranged from 0·03 to 0·3% with 50 to 200 µg of N, from 0·04 to 0·3% with 20 µg of N, and from 0·3 to 0·7% with 10 µg of N.

### Acknowledgement

This work was a part of Project ILLU-15-0392, Illinois Agricultural Experimental Station, College of Agriculture, University of Illinois, Urbana, Illinois, USA.

### References

1. Barrie, A., Brookes, S. J. and Debney, S., *Fertilizer Research*, **42** (1995), 43.

2. Barrie, A. and Prosser, S. J., in *Mass Spectrometry of Soils*, Boutton, T. B. and Yamasaki, S. (Eds), (Marcel Dekker, New York, 1996), 1.

3. McInteer, B. B. and Montoya, J. G., in *Recent Developments in Mass Spectrometry in Biochemistry, Medicine and Environmental Research*, Frigerio, A. (Ed), (Elsevier, Amsterdam, 1981), 343.

4. McInteer, B. B., Montoya, J. G. and Stark, E. E., *Spectroscopy International Journal*, **3** (1984), 226.

5. Mulvaney, R. L., Fohringer, C. L., Bojan, V. J., Michlik, M. M. and Herzog, L. F., *Review of Scientific Instruments*, **61** (1990), 897.

6. Mulvaney, R. L. and Liu, Y. P., *Journal of Automatic Chemistry*, **13** (1991), 273.

7. Sprinson, D. B. and Rittenberg, D., *U.S. Naval Medical Bulletin*, March-April Supplement (1948), 82.

8. Mulvaney, R. L., in *Nitrogen Isotope Techniques*, Knowles, R. and Blackburn, T. H. (Eds), (Academic Press, San Diego, 1993), 11.

9. Bremner, J. M., in *Methods of Soil Analysis*, Part 3, Sparks, D. L. *et al.* (Eds), (Soil Science Society of America, Madison, Wisconsin, 1996), 1085.

10. Hauck, R. D., in *Methods of Soil Analysis*, Part 2, 2nd edn, Page, A. L. *et al.* (Eds), (American Society of Agronomy, Madison, Wisconsin, 1982), 735.

11. Mulvaney, R. L., Khan, S. A., Stevens, W. B. and Mulvaney, C. S., *Biology and Fertility of Soils*, **24** (1997), 413.

12. Khan, S. A., Mulvaney, R. L. and Mulvaney, C. S., *Soil Science Society of America Journal*, **61** (1997), 936.

13. Brooks, P. D., Stark, J. M., McInteer, B. B. and Preston, T., *Soil Science Society of America Journal*, **53** (1989), 1707.

14. Sørenson, P. and Jensen, E. S., *Analytica Chimica Acta*, **252** (1991), 201.

15. Goswami, D., *Heating Piping Air Conditioning*, **65** (8) (1993), 75.

